# Supplementary material for: High Mobility Group AT-Hook 2 (HMGA2) Oncogenicity in Mesenchymal and Epithelial Neoplasia
Source: Int J Mol Sci. 2020 Apr 29;21(9):3151. doi: 10.3390/ijms21093151 (PMC7246488; doi:10.3390/ijms21093151)
Supplement: Supplementary file 1 [file ijms-21-03151-s001.pdf]

Supplementary Table S1: Chromosomal rearrangements involving HMGA2 in human mesenchymal tumors.

Tumor Type	[Refs]	Chromosomal Translocation Partners	Chromosomal Rearrangement
Lipoma	[1]	1	t(1;12)(p36;q15)
	[1]		t(1;12)(p36;q14-15)
	[1]		t(1;12)(p34;q15)
	[1]		ins(12;1)(q15;p33p22)
	[1]		t(1;12;2)(p33;q15;q37)
	[1]		**t(1;12)(p33;q15),**t(3;5)(p23;q13)
	[1]		t(1;12)(p33;q15)
	[1]		t(1;12)(p32;q15)
	[1]		**t(1;12)(p32;q13),**t(6;19;8)(q13;q13;p21),der(10)t(10;11)(p13;q21), der(11)t(11;16)(q21;q13),der(16)t(16;17)(p11;p11)del(16)(q13), der(17)t(16;17)(p11;p11)
	[1]		**[t(1;12)(p36.1;q13)]**,t(der(1);5;15)(der(1)(15qter→15q24::12q15→12q13::1p36.1→cen→1qter),der(5)(5pter →cen→5q33::12q15→12qter),der(15)(15pter→cen→15q24::5q33→5qter))
	[1-3]		t(1;12)(p32;q14)
	[1]		t(1;12)(p22;q14)
	[1]		ins(12;1)(q13;p12p22)
	[1]		der(1)ins(1;12)(q21;q21q13)t(1;12)(q32;q21), der(12)t(1;12)(q32;q13)
	[1]		t(1;12)(q23;q15)
	[1]		t(1;12)(q32;q14)
	[1]		der(1)inv(1)(p22q42)**t(1;12)(q44;q15)**,?t(3;4)(p25;p13),**del(12)(q13)**
	[1]		**der(1)t(1;12)(q42;q15), der(12)t(12;14)(q15;q21)**t(1;14)(q42;q24),del(14) (q21q24)
	[4]		t(1;12)(p33;q14)
			t(1;12)(p32;q13q15)
	[3]		t(1;12)(p32;q14)
	[3]		t(1;12)(p32;q14),del(13)(q14q31)
	[3]		t(1;12)(p32;q14),t(1;20)(q32;p13)
	[3]		t(1;12)(p32;q14),t(11;12)(q13;p13)
	[3]		t(1;12)(p32;q14), del(1)(p32),t(2;11)(q21;p15)
	[3]		inv(1)(p32q11),t(1;12)(q11;q14),t(17;18)(q11;q23)
	[4]		-1,+der(1)ins(1;?)(p21;?),-12,**+der(12)ins(12;?)(q13;?)**
	[4]		-1,+der(1)ins(1;12)(p32;q13q15),-12,+der(12)(12qter→12q24.3::12q15→12q24.3::12p11.2→cen→12q13::12p11.2→12pter)
	[4]	2	t(2;12)(p21-22;q13-14)
	[1]		t(2;12)(p21;q15)
	[4]		t(2;12)(q11.2;p11.2),t(19;20)(q13.1;p13)
			t(2;12)(q11.2;q15 or 21),del(13)(q12q22)
	[4]		del(1)(q31),t(2;12),t(3;11),+r
	[1]		der(2)t(2;6)(q33;q12),der(6)t(6;12)(q12;q11)**ins(12;2)(q13;q33q37) ,del(12) (q11)/46,idem,t(7;12)(p15;q24)**

	[1]		t(2;12)(q35;q15)
	[1]		t(2;12)(q35;q15)/46,XY, der(1)t(1;12)(p36;q13)t(2;12), der(2)t(2;12), der(12)t(1;12)
	[1]		**t(2;12)(q35;q15)**,der(4)t(4;20)(q28;q11), der(11)t(11;13)(p11;q14)t(1;11)(p34;q13), der(13)t(4;13)(q28;q14), der(17)t(11;17)(q23;p11)del(11)(q13),der(20)t(17;20)(p11;q11)
	[1]		**t(2;12)(q35;q15)**,der(3)inv(3)(p21q12)add(3)(p21)add(3)(q23)del(3)(q12q23),der(6)t(6;13)(q14;p11), -13,der(17)t(3;17)(p26;q23), der(20)t(17;20)(q23;p13)
	[1]		t(2;12)(q36;q14)
	[1]		t(2;12)(q37;q13)
	[5, 6]		t(2;12)(q37;q14)
	[1]		der(2)t(2;10)(q13;q24),der(7)t(2;7)(q13;q36)**t(2;12)(q37;q15) ,add(12)(q15)**
	[1, 4, 7-12]	3	t(3;12)(q28;q14)
	[1]	3	t(2;3)(p21;q29),**t(3;12)(p14;q15)**
	[1]	3	der(2)t(2;3)(p23;p21),der(3)inv(3)(p13p21)t(3;12)(p13;q15) ,**der(12)t(2;12)(p23;q15)**
	[1]	3	der(3)ins(3;12)(q13-21;q12q14)del(3)(q13-21q27),der(12)ins(12;3)(q12;q13-21q27) del(12)(q12q14)
	[1]	3	ins(12;3)(q15;q13-21q27-28)
	[1]	3	ins(12;3)(q15;q23q25)/46,idem,ins(1;10)(p12;q11q25)
	[1]	3	t(3;12)(q25;q15)
	[1]	3	t(3;12)(q26;q15)
	[1]	3	t(3;12)(q27;q15)
	[1, 11]	3	t(3;12)(q27;q13)
	[2]	3	t(3;12)(q27;q13-q15)
	[1]	3	t(3;12)(q28;q13-q15)
	[4]	3	t(3;12)(q28;q14),t(3;17)(q12;q11.2)
	[1, 4]	3	t(3;12)(q27;q14)
	[1]	3	t(1;7)(q25;q22),**t(3;12)(q27;q14)**
	[1]	3	**t(3;12)(q27;q14),**t(10;17;17)(q24;p11;p13)
	[1]	3	der(3)ins(3)(p21q25q27)**t(3;12)(q27;q15) ,der(12)t(3;12)**
	[1]	3	t(3;8)(q13;q22),**t(3;12)(q27;q15)**
	[1]	3	**t(3;12)(q27;q14)/** del(1)(p32), **der(3)t(3;12), der(12)t(3;12) add(12)(p12),+22**
	[1]	3	der(3)t(3;9)(p21;q34)**t(3;12)(q27;q15)** ,t(7;15)(q22;q24),der(9)t(3;9), **der(12)t(3;12) ,del(13)(q12q31)**
	[1]	3	der(3)t(3;11)(p14;q21)**t(3;12)(q27;q15)** ,der(11)t(3;11),**der(12)t(3;12)**
	[1]	3	**t(3;12)(q27;q15)/** idem,t(1;11)(p36;q23),t(4;20)(p14;p11)
	[1]	3	der(3)t(3;10)(p21;q11)t(3;20)(q27;q11),der(10)t(3;10), **der(12)t(3;12)(q27;q14)** ,der(20)t(12;20)(q14;q11) /idem,**t(2;12)(p24;q15)**
	[1]	3	t(3;12)(q27-28;q13-15)
	[1]	3	t(3;12)(q27-28;q14-15)
	[1]	3	t(3;12)(q28;q15)

	[1]	3	t(3;12)(q28;q13)
	[1]	3	**t(3;12)(q28;q15)/**46,idem,t(1;6)(p36;q25)
	[1]	3	**t(3;12)(q28;q14)/**47,idem,+5
	[1]	3	der(3)inv(3)(p21q11)**t(3;12)(q28;q14)** ,t(5;15)(q14;p11), **der(12)t(3;12)(q28;q14)** ,add(13)(q12) ,der(16)t(13;16)(q13;p11) /add(3)(q29),der(3)inv(3)del(3)(q2?1q2?8)**t(3;12) ,t(5;15),der(12)t(3;12)**,add(13) , der(16)t(13;16)
	[1]	3	t(3;12)(q29;q14)
	[4]	3	**[inv(12)(p11.2q13)]**,+t(3;inv(12))(der(3)(12pter→12p11.2::12q15→12q13::3p21→cen→3qter);der(12)(12qter →12q15::12p11.2→cen→12q13::3p21→3pter))
	[11]	3	dup(3)(p26p25),**t(3;12)(q27;q14)**,dup(22)(q11q11)
	[4]	4	Ins(4;12)(q21;q11q15)
	[4]	4	-4,+der(4)t(4;12)(4p16→cen→4q22::12q14→12qter),-12,+der(12)t(4;12)(4qter→4q28::12p13→cen→12q14::4q22→4q28::12p13→12pter)
	[1]	4	ins(4;12)(p16;q24q15) ,+9
	[1]	4	**der(4)t(4;12)(q24;q14)** ,der(5)ins(5;4)(q13;q24q27), **der(12)t(4;12)(q27;q14)**
	[1]	4	t(4;12)(q27;q15)
	[1, 2, 11, 13]	5	t(5;12)(q33;q15)
	[13]	5	t(3;7)(p13;p15),**t(5;12)(q33;q14)**
	[13]	5	**t(5;12)(q33;q14)**/idem,der(14;21)(q10;q10)
	[13]	5	t(5;12)(q33;q14)
	[11]	5	del(2)(p23p24),dup(2)(q13),**t(5;12)(q33;q14)**,del(21)(q21)/ idem,t(1;6)(p36;p23)
	[1, 13]	5	ins(5;12)(q33;q14q21)
	[1, 11, 13]	5	t(5;12)(q33;q15)
	[13]	5	t(5;12)(q32;q14)
	[13]	5	t(5;12)(q32;q15)
	[1, 13]	5	ins(12;5)(q15;q33q13)
	[4, 13]	5	t(5;12)(q33;q14)
	[2, 13]	5	ins(12;5)(q14;q33q13)
	[1]	5	**t(5;12)(p13;q15)**, del(13)(q14)
	[1]	5	**t(5;12)(q11;q15)**,add(13)(q12),?del(22)(q12)
	[1]	5	?inv(5)(q14q22),**t(5;12)(q31;q14)**
	[1, 13]	5	**t(5;12)(q32;q14)**/idem, add(2)(p14),add(4)(q21),der(18)t(4;18)(q21;q?21)
	[1]	5	t(3;7)(p13;p15),**t(5;12)(q33;q14)**
	[1]	5	**t(5;12)(q33;q14)**/ idem,der(14;21)(q10;q10)
	[2]	5	t(5;12)(q32-33;q14-15)
	[1]	5	**t(5;12)(q33;q14)**/ idem,t(1;6)(p36;p23)
	[1]	5	-1,**t(5;12)(q33;q15)**, t(8;12)(q21;p11), t(9;15)(p13;q26),del(13)(q22q31) , der(20)t(1;20)(p32;q11)/idem,+r(1)(p22q44)
	[1]	5	t(5;12)(q34;q15)
	[1]	5	t(5;12)(q34;q14-15)
	[4]	6	Ins(6;12)(p23;q15q21)

	[1]	6	t(6;12)(p21;q14)
	[1]	6	der(2)t(2;?12)(q3?7;q11)t(?6;12)(p21;q15) ,add(6)(p21) ,-12,+der(?)t(?;12)(?;q15)
	[1]	6	del(1)(p13p22),**der(6)t(6;12)(p21-22;q15) ,add(12)(q15)**
	[1]	6	t(6;12)(q21;q15)
	[1]	6	der(3)t(3;6)(q23;q21)**t(6;12)(q27;q14)**,del(6)(q21),**der(12)t(3;12)(q23;q14)**
	[1]	7	dic(7;12)(p21;q15),-12,der(22)ins(22;7)(q13;p21p22),+1-2mar
	[1]	7	t(5;15)(p14;q14),**t(7;12)(p14;q15)**,del(15)(q24)
	[1]	7	der(4)t(4;7)(p15;p13),del(6)(q15),**der(7)t(7;12)(p13;q13)t(?6;12)(q?21;q?21)**,del(12)(q13)
	[1]	7	**der(7)t(7;12)(q31;q14)**,t(8;22)(p23;q11),**der(12)t(7;12)(q31;p13),del(12)(q14)**
	[9]	8	t(8;12)(p22;q14;q22.3-qter)
	[9]	8	t(8;12)(p22;q14;q22)
	[1]	8	der(1)t(1;13)(q21;q14),der(8)t(1;8)(q21;p21),<br>**der(12)t(8;12)(p21;p13)**,add(13)(q11)
	[1]	8	t(8;12)(q13;q15)
	[1]	8	ins(12;8)(q15;q24q24)
	[1]	8	ins(12;8)(q24;q13q22)
	[14-17]	9	t(9;12)(p22;q14)
	[1]	9	ins(9;12)(p24;q15q23)/47,idem,+der(9)ins(9;12)
	[1]	9	t(9;12)(p22;q15)
	[1]	9	add(6)(q15),**der(9)t(9;12)(q22;q15)**,del(10)(q22),der(11)t(11;14)(q23;q24),<br>**der(12)t(10;12)(q22;q15)** ,add(13)(p11),add(14)(q24),-17
	[1]	9	**t(9;12)(q33;q14)**/ -9,der(11)t(9;11)(q3?1;q?21)**t(9;12)(q33;q14)**,?der(12)t(9;12),+mar
	[4]	10	t(10;12)(p11.2;q14 or 15)
	[1]	10	t(10;12)(p15;q15)/idem, +der(12)t(10;12)
	[1]	10	t(10;12)(p11;q13)
	[1]	10	t(10;12)(q25;q15)
	[1]	10	t(10;12)(q22;q14)
	[1, 2]	10	t(10;12)(q22;q15)
	[1, 4]	11	t(11;12)(q13;q14)
	[1]	11	add(10)(q23),**der(11)ins(11;12)(q11;q13q21)**del(11)(q11q25),<br>**der(12)t(11;12)(q11;q24)del(12)(q13q21) add(12)(p11)**
	[1]	11	**ins(11;12)(q13;q24q15)**/ idem,del(17)(q23)
	[1]	11	der(6)t(6;13)(p21;q12),der(10)t(10;11)(q24;q13), **der(11)t(11;12)(q13;q13)**,<br>der(11)t(11;14)(q13;q24), **der(12)t(10;12)(q24;q13)**,der(14)t(6;14)(p21;q24),<br>+der(?)t(?;11)(?;q13)/46,idem, del(13)(q22)/46,idem,t(1;16)(p11;p11)
	[1]	11	t(11;12)(q21;q14)
	[1]	11	der(11)**t(11;12)(q11;p12),der(12)del(12)(q13q15)inv(12)(p12q13)t(12;15)**<br>**(q13;q12)** ,der(15)t(11;15)(q11;q12)
	[4, 9]	Intragenic	-12,+der(12)(12qter→12q24.1::12p13→cen→12q13::12q15→12q13::12q15→12q24.1::12p13→12pter)
	[1]	Intragenic	inv(12)(q13q15)
	[4]	Intragenic	del(12)(q13q15 or q21)
	[1]	Intragenic	del(12)(q14q21)
	[1]	Intragenic	der(12)t(12;?12)(p13;q15)
	[1]	Intragenic	inv(12)(p12q15)
	[1]	Intragenic	**inv(12)(p12q14)**/idem,tas(19;21)(p13;p13)

	[1]	Intragenic	der(12)inv(12)(p13q24)inv(12)(p12q15)
	[1]	Intragenic	inv(12)(p11q15)
	[1]	Intragenic	add(8)(p?11),**add(12)(q1?3)**
	[1]	Intragenic	der(12)ins(12;?)(q13;?)dup(12)(q13q22)
	[1]	Intragenic	**der(12)?ins(12;?)(q13;?)?del(12)(q23)**,del(15)(q12),der(17)t(15;17)(q22;q25)
	[1]	Intragenic	t(1;1)(p13;q42),**del(12)**
	[1]	Intragenic	dup(12)(q13→q22)
	[1]	Intragenic	t(12;12)(q14;q23)
	[1]	Intragenic	der(12)t(12;16;?)(q14-q15;q22;?)
	[1]	Intragenic	Inv(12)(p12q13-q14)
	[4]	Intragenic	[inv(12)(p11.2q13)],+t(3;inv(12))(der(3)(12pter→12p11.2::12q15→12q13::3p21→cen→3qter);der(12)(12qter →12q15::12p11.2→cen→12q13::3p21→3pter))
	[1]	Intragenic	del(12)(q14q21)
	[1]	Intragenic	ins(12;12)(q15;q13q15)
	[1]	Intragenic	+8,+der(12)del(12)(p11p12)del(12)(q13q15)
	[1]	Intragenic	del(4)(q27),der(12)(q13q15)inv(12)(q15q24) t(4;12)(q27;q24)
	[1]	Intragenic	der(8)t(8;15)(q22;q22),del(12)(q15) ,der(14)t(12;14)(q15;q22), add(15)(q22),der(17)t(14;17)(q22;p13)
	[1]	Intragenic	add(12)(q15), +der(?)t(?;12)(?;q15)/49-50,XY,-12,+der(?)t(?;12)(?;q15), +2mar,inc
	[1]	Intragenic	t(7;18)(q21;q22),**ins(12;?)(q15;?)**
	[1]	Intragenic	der(1)t(1;12)(p32;p13),der(12)inv(12)(q13q15) t(1;12)(p32;p13)/idem,+7,+19
	[1]	Intragenic	inv(12)(q13q21)
	[2]	Intragenic	inv(12)(q14q24)
	[1]	Intragenic	t(4;13)(q?21;q?14), **?inv(12)(q14q21)**/del(6)(q23), **?inv(12)**
	[1]	Intragenic	der(10)t(10;12)(p11;q24),der(12)inv(12)(q13q22) t(10;12)
	[1]	Intragenic	der(12)del(12)(q13q13-14)inv(12)(q13-14q23)
	[1]	Intragenic	?der(5)t(5;21)(q11;q21),**?der(12)ins(12;5)(p13;q35q11)del(12)(q13q23) , ?der(21)t(12;21)(q15;q21) del(12)(q23)**
	[1]	Intragenic	inv(12)(q15q24)
	[1]	Intragenic	der(1)inv(1)(p32q25)t(1;9)(p32;q22),der(9)t(1;9),inv(12)(q15q24)
	[1]	Intragenic	ins(12;12)(q24;q13q24)/46,idem,t(1;6)(p22;q12)
	[18]	Intragenic	der(12)ins(12;12)(pl 1;q13-14q15)
	[18]	Intragenic	der(12)inv(1 2)(p13p1 1)inv(1 2)(q14q24)
	[18]	Intragenic	inv(12)(pl1.2q15)
	[1]	Intragenic	add(12)(q24)
	[19]	Intragenic	+r(12)
	[20]	Intragenic	t(12;12)(q14;q14)
	[1]	13	t(12;13)(q13;q12)
	[1]	13	t(12;13)(q15;q14)
	[1]	13	der(12)t(12;13)(q15;q21-22), der(13)t(12;13)(q15;q14)
	[11]	13	t(12;13)(q24;q14)
	[11]	13	t(12;13)(q22;q14)
	[11]	13	**del(12)(q14q14),ins(13;12)(q22;q21q23)**,-del(13)(q31q31)/45,idem,_21/t(13;14)(q14;q32)
	[21]	13	t(12;13)(q14;q13)
	[1]	13	der(12)t(12;13)(q15;q22),der(13)t(12;13)(q15;q14)

	[1]	13	t(12;13)(q15;q22)
	[1]	13	t(12;13)(q15;q34)
	[10]	13	t(12;13)(q14;q21-32)
	[1]	14	t(12;14)(q13;q24)
	[1]	14	t(12;14)(q15;q22)
	[9]	14	t(12;14)(q14;q14)
	[10]	15	t(12;15)(q14;q24)
	[1]	16	t(12;16)(q15;p11)
	[1]	16	t(12;16)(q15;q22)
	[4]	17	Inv(7)(p13q22),**t(12;17)**
	[4]	17	t(12;17)(q14;p13)
	[4]	17	Inv(7),**t(12;17)**,t(13;15)(p11;q11)
	[1]	17	der(9)t(9;17)(q32;q23),**der(12)t(12;17)(q14;p12) ,der(17)t(12;17)**t(9;17)
	[1]	17	t(12;17)(q13;p11)
	[1]	17	ins(17;12)(q21-23;q13q14-15)
	[1]	18	t(12;18)(q13;q11)
	[20]	18	t(12;18)(q14;q12)
	[1]	18	der(12)add(12)(p13)t(12;18)(q13;q21) ,?der(18)t(12;18)(q13;q12)
	[1]	18	inv(5)(p15q21),**der(12)t(12;18)(p13;q21)t(12;17)(q15;q23) ,der(17)t(12;17) , der(18)t(12;18)**
	[20]	18	t(12;18)(q14~q15;q12~q21)
	[20]	18	t(12;18)(q14~q15;q12~q21)
	[20]	18	t(12;18)(q14~q15;q12~q21)
	[20]	18	t(12;18)(q14~q15;q12~q21)
	[1]	20	**t(12;20)(q15;p13)**/idem,inv(1)(q24q41)
	[1]	20	**t(12;20)(q13;q11)**, del(13)(q12q22-31)
	[1]	20	t(12;20)(q15;q11)
	[4]	21	t(12;21)(q13;q21)
	[4]	21	t(12;21)(q12 or 13;q21)
	[1]	21	t(12;21)(q15;p11)
	[1]	21	ins(21;12)(q21;q13q15)
	[1]	21	**t(12;21)(q14;q22)/** idem,add(16)(p?)/idem,der(2)t(2;7)(p23;q32), der(7)t(2;7)(p23;q11)
	[1]	22	t(1;12)(p32;q24)/46,XY,der(1)t(1;12)(p32;q24),**der(12)t(12;22)(q15;q12) , der(22)t(12;22) t(1;12)**
	[1]	22	t(1;12)(p32;q24)/46,XY,der(1)t(1;12)(p32;q24),**der(12)t(12;22)(q15;q12) , der(22)t(12;22) t(1;12)**
	[4]	X	t(X;12)(q27;q14)<br>t(X;12) with random abnormalities
	[1]	X	**der(X)t(X;12)(p22;q14)**,der(11)t(11;17)(p11;q21)ins(11;?)(p11;?), **der(12)add(12)(p11) t(12;17)(q14;q12)**,add(17)(q11-12),del(17)(q12)
	[1]	X	t(X;12)(q24;q24)
Lipoma (w/ >1 Chromosomal Partners)	[9]	7, 13	t(7;13;12)(p16-p21;q14-q21;q13)
	[1]	2, 5, 18	t(2;12;5;18)(p23;q15;q13;q12)
	[3]	1, 19	t(1;12;19)(p32;q13;q11)

	[4]	2, 5, 10	t(2;5;5;10;12)
	[4]	1, 9	[Inv(12)(p13.3q24.1)],t(1;9;inv(12))(der(1)(12pter→12p13.3::12q24.1→12q13.1::1p36.2→cen→1qter);der(9)(9pter→cen→9q31::1p36.2→1pter);der(12)(12qter→12q24.1::12p13.3→cen→12q13.1::9q31→9qter))
	[4]	1, 5, 7, 9	-1,+der(1),-5,+der(5),-7,+der(7),-9,+der(9),-12,**+der(12)**
	[1]	1, 4	**der(1)t(1;2)(p13;q13)t(1;12)(q32;q15)**,der(2)t(1;2)(p13;q13),add(4)(q21),**der(12)?t(1;12)(q32;p13) t(4;12)(q21;q15),**?add(15)(q22)
	[1]	1, 14	der(1)**t(1;12)(q42;q15),der(12)t(12;14)(q15;q21)**t(1;14)(q42;q24),del(14)(q21q24)
	[1]	2, 5, 3	t(2;12;5;3)(q11;q13;q33;p14)
	[1]	2, 3, 21	**t(2;12;21;3)(q33;q15;q11;p13)**,t(5;22)(q13;q13)
	[1]	3, 13	t(3;12;13)(q28;q14;q14)
	[1]	2, 3	**t(2;3;12)(p24;q28;q15)**/idem,del(3)(q11q13)
	[1]	1, 3, 4	t(1;4;12)(p32;q27;q15), **t(3;12)(p23;q15)**,t(5;9)(q31;q32)
	[1]	6, 11	**t(6;12;11)(p12;q14;p15)**/idem,der(13;15)(q10;q10)
	[1]	3, 6	t(3;6;12)(q21;q23;q15)
	[1]	7, 17	**t(7;12;17)(p15;q14;q25)**,inv(17)(q21q24)
	[1]	3, 8	t(3;8;12)(p11;p21;q13)
	[1]	2, 9	?t(2;9;12)(p23;q34;q15),t(3;15)(q21;q22)
	[1]	2, 16	t(2;16;12)(p13;q24;q15)
	[1]	13, 18	t(12;13;18)(q15;q14;q21)
	[1]	10, 20	t(1;7)(q32;p22),**t(10;20;12)(q22;q11;q15)/t(10;20;12)**,t(15;17)(q15;p13)
	[1]	1, 16, 21	t(1;16;21;12)(p36;q22;q22;q15)
Osteochondrolipoma	[20]	18	t(12;18)(q14~q15;q12~q21
Uterine Leiomyoma	[22]	1	t(1;12)(q32;q14)
	[23]	5	4,-5 ,-14,inv(6)(p25q21),t(8;14)(q24;q24),**inv(12)(q15q24.1), +der(12)ins(12;5) (q15;q12q35)**,del(13)(q22q32),+mar
	[24]	7	t(7;12)(q31;q14)
	[25, 26]	8	t(8;12)(q22;q14)
	[9]	8	t(8;12)(q22.3;q14)
	[27]	9	r(1),add(2)(p13),add(4)(q21),-8,-9,der(9)**t(9;12)(q34;q12)r(9;12)(p22;q24)**, del(11)(q23q25),-12,+4mar
	[9]	11	Ins(12;11)(q14;q21qter)
	[27]	intragenic	der(2)t(2;14)(q35-36;q24),add(5)(q31),del(7)(q22q32),**add(12)(q13–15)**, -14,add(17)(q25),inc/38–48,idem,r(1)(p32–34q44)
	[28]	Intragenic	der(12)(q14)
	[22]	14	t(12;14)(q14;q32)
	[9]	14	t(12;14)(q15;q24)
	[9]	14	t(12;14)(q14-15;q24)
	[27, 29, 30]	14	t(12;14)(q14;q24)
	[31]	14	t(12;14)(q15;q11)
	[9]	14	t(12;14)(q16;q24)
	[32, 33]	14	del(1)(q42),**t(12;14)(q14;q24)**
	[32]	14	der(14)**t(12;14)(q14–15;q23–24)**, der(22)(pter = q11::?)
	[29, 32]	14	t(12;14)(q14–15;q23–24)
	[22]	14	t(12;14)(q14;q23–24)

	[32]	14	t(12;14)(q14–15;q23-24),der(1)t(1;2)(p32;q22),-2,t(12;14)(q14–15;q23–24),-13,der(21)t(13;21)(q12;q22)
	[32]	14	t(12;14)(q13;q32). ish der(12)del(12)(q15)t(12;14)(q13; q32)(HMGIC-,wpc12+, wpc14+),der(14)ins(14;12)(q23–24;q15)t(12;14)(q13;q32)(HMGIC+,wpc12+, wpc14+)
	[32]	14	der(1;2)(q?;q?),-10,**t(12;14)(q15;q23–24)**,-14,add(21)(p11),+ma r1,+mar2 cp
	[33]	14	r(1),t(4;6)(p16;q21), der(12)inv(12)(p13q11)ins(12)**t(12;14)(q15;q21q24)**, del(14)(q21q24)
	[23]	14	r(1)(p34q32),ins(8;9)(q13;q13q22),**t(12;14)(q14–15;q23–24)**
	[32]	14	der(1;2)(q?;q?),-10,**t(12;14)(q15;q23–24)**,-14,add(21)(p11),+ma r1,+mar2 cp
	[34]	14	del(7)(q22q32),**t(12;14)(q15;q24)**
	[27]	14	add(3)(p21),der(4)t(3;4)(p21;p12),**t(12;14)(q14–15;q23–24)**
	[27]	14	r(?1)(?p32q21),**t(12;14)(q14–15;q23–24)**,der(16)(q12)
	[23]	14	+5,+5,+6,+6,+7,+7,+8,+8,+9,+9,+ 10,+ 10,+19,+19,+20,+20,+21,+21,+22,+22,+t(1;?)(p36;?),+t(l;?) (p36;?), +del(3) (q13q26),+del(3)(q13q26),+t(ll;17) (ql3;q21),+t(11~ 17)(q13;q21), +**t(12;14)(q14-15;q23-24), +t(12;14)(q14-15;q23-24)**,t(14;?)(q24;?),t(14;?)(q24;?), +t(15;?)(q26;?),+t(15;?)(q26;?),+ der(17)t(ll;17) (q13;q21), +4mar
	[23]	16	r(1),der(2)t(1;2)(q21;p23q13),der(2)t(2;9)(p21;q13),add(5)(q35)*2,+der(5) t(5;15)(q11;q15),-6,-7 ,der(8)t(8;11)(q24;q13),-10,-11,add(15)(p12),der(16)**t(12;16)(q13;p13)**,+20,-22,+r,+mar
	[9]	X	t(X;12)(q22;q15)
	[9]	2, 3	t(2;3;12)(q35;p21;q14)
	[34]	2, 3	t(2;3;12)(q35;p21;q14)
Lipoblastoma	[35]	4	t(4;12)(p16;q14)
Soft Tissue Chondroma	[36]	3	t(3;12)(q27;q15)
		4	ins(4;12)(q3?4;q14q2?3)
		17	add(12)(q13),der(17)t(12;17)(q13;q21)
		8, X	der(8)t(8;12)(q11;q15)del(8)(p22),der(12)t(X;12)(q24;q15)
Skeletal Chondroma	[36]	Intragenic	inv(12)(p12q13)
Skeletal Chondrosarcoma	[36]	13	t(12;13)(q13;p13)
		1	t(1;12)(p36;q13)
		Intragenic	+der(?)t(?;12)(?;q13)_2
		14	t(12;14)(q13;q24)
		1	der(1)t(1;12)(p36;q13)
Inflammatory myofibroblastic tumor/myofibrobl astic sarcoma		4	der(4)?t(4;12)(q23;q15),add(10)(q26),der(12)?inv(12)(q?q?)add(12)(q?q15), der(21)t(4;21)(q23;q22),1mar.ishder(4)?t(4;12)(q23;q15)(pcp27E12–142H11),add(10)(q26)(pcp27E12–142H11),der(12)?inv(12)(q?q?)add(12)(q?q15)(pcp27E12–142H12),der(21)t(4;21)(q23;q22),1mar
Idiopathic Myelofibrosis	[37]	4	t(4;12)(q32;q15)
		5	t(5;12)(p14;q15)
Inflammatory Myofibroblastic Tumor	[38]	Intragenic	add(12)(q14)

De-differentiated Liposarcoma	[39]	16	+r(12;16)
Sarcoma	[40]	Intragenic	t(12;12)(q15;q14)
	[40]	1	t(1;12)(p32;q14)
	[40]	Intragenic	t(12;12)(q14;q13)
	[40]	Intragenic	t(12;12)(q14;q12)
	[40]	Intragenic	t(12;12)(q14;q21)
	[40]	1	t(1;12)(p32;q14)
	[40]	Intragenic	t(12;12)(q14;q13)
	[40]	11	t(11;12)(p11;q14)
	[40]	Intragenic	t(12;12)(q14;q22)
	[40]	6	t(6;12)(q24;q14)
Myolipoma	[41]	9	t(9;12)(p22;q14)
Aggressive angiomyxoma	[18, 42]	5	der(5)t(5;12)(q31;p11.2),der(12)t(5;12)(q31;p11.2)inv(12)(p11.2q15)
	[43]	Intragenic	der(12)(q14)
	[44]	21	t(12;21)(q15;q21.1).isht(12;21)
	[45]	8	t(8;12)(p12;q15)
	[46]	1	t(1;12)(p32;q15)
Hyaline Vascular Castleman's Disease (HVCD)	[47]	6	add(1)(q21),**der(6)t(6;12) (q23;q15)**,add(7)(p22), −9,inv(9)(p11q13),**del(12)(q15)**,+mar
Synovia from Osteoarthritis	[48]	13	t(1;14)(q25–27;q13–22),t(2;7)(q23;q36),**t(12;13)(q15;q32)**
	[48]	X	**t(X;12)(q26;q15)**,add(10)(p11)
	[48]	Intragenic	inv(12)(p11q13)
Extra-skeletal Osteochondroma	[49]	5	der(5)t(5;12) (q35;q14~15),der(12)t(5;12)inv(12)(p11q14~15)
	[50]	Intragenic	inv(12)(qter->q14~15::p11->q13::q14~15->q13::p11->pter) [13]/idem,t(5;13)(q13;p11)
Periosteal Osteosarcoma	[19]	Intragenic	+r(12)
Spindle Cell Sarcoma	[51]	Intragenic	del(X)(p?11p?22), **der(12)(12pter→12q?22::12q?15→q?22::16p11→ 16pter),-16,+r(12)**.
Chondrolipoangioma	[52]	2, 15	t(2;12;15) (q37;q13;q26)
Pulmonary Chondroid Harmatoma (PCH)	[18]	Intragenic	inv(12)(p1.2q15)
	[53]	1	der(1)t(1;13)(p22;q32)**ins(1;12)(p22;q24.3q15), del(12)(q15)**,der(13)t(1;13)(p22;q32)
		1	del(8)(q22),**ins(12;1)(q15;p23p13)**,der(15)t(8;15)(q22;q11.2 or q12),ins(17;15)(q21;q11.2 or q12q23)
		1	der(1)t(1;12),der(12)?inv(12)t(1;12)
		2	XY,der(2)?**t(2;12)(p25;q15)**,del(6)(q15q21), **der(12)del(12)(p12)add(12)(q15)**

		2	der(2)t(2;7)(q33;q36), **der(2)t(2;12)(p23;q15),**der(7)t(2;7)(p23;q36), **der(12) t(2;12)(q33;q15)**
		3	ins(12;3)(q15;q13.2q29)
		3	t(3;12)(q27;q15),der(10)add(10)(p15)t(10;21)(q21;q21), der(21)t(10;21) (q21;q21)
		3	**t(3;12)(q29;q15)**/idem,del(1)(q2?),der(3)add(3)(p21)dup(3)(q23q29)
		3	t(3;12)(q29;q15)
		3	t(3;12)(q27;q15)
		3	add(X)(q13),**der(3)t(3;12;X),der(12)t(3;12)(q27;q15)**
		3	ins(3;12)(q27 or q28;q12q21)
		3	t(3;12)(q12;q15)
		3	t(3;12)(q27 or q28;q15)
	[54]	3	t(3;12)(q27;q14-q15)
	[53]	3	t(3;12)(q24 or q25;q15)
		4	der(4)t(4;12)(q35;q14),del(12)(q14)
		4	t(4;12)(q12;q15)
		4	t(4;12)(p14;q15)
		4	t(4;12)(p15.1 or 15.2;q15)
		5	del(5)(q22),add(6)(q27),**der(12)inv(12)(q13q15)t(5;12)(q22;q13)**
		5	del(5)(q31),**t(5;12)(q31;q15)inv(12)(q15q24.3)**,del(16)(q21q23)
		5	X,?t(X;4),**t(5;12)(q14 or q15;q15)**
		5	t(5;12)(q34;q14 or q15)
		6	t(6;12)(q25;q14)
		6	t(6;12)(p21.3;q22)
		6	**t(6;12)(q22.2;q15)**,t(11;17)(q21;q23)
		7	**t(7;12)(p22;q15)**,der(10)t(10;14)(q24;q22),del(14)(q22)
		7	**der(7)t(7;12)(p13;p11.2)ins(7;14)(p13;q24q11.2), der(12)del(12)(q14q21)t(7;12) (p13;p11.2),**del(14)(q11.2q24)
		8	ins(12;8)(q15;p22p23)
		8	t(3;19)(q13;q13.2 or q13.3),**der(8)t(8;12)(q22;q15), add(12)(q15)**,del(15)(q22)
		8	del(8)(p21),**der(12)inv(12)t(8;12)**
		9	t(9;12)(p23;q15)
		10	ins(12;10)(q15;q11.2q26)
		10	der(10)t(10;12)(p15;p13.1), der(12)del(12)(p13.1)inv(12)(p13.1q15)
		11	t(11;12)(p15;q15)
		11	der(4)t(4;12)(q35;q15),der(7)del(7)(p13)del(7)(q32), der(9)t(7;9)(p13;q34), der(11)t(7;11)(q32;q23), **der(12)ins(12)(p13q13q15)t(11;12)(q32;q13)**
		11	t(11;12)(q13;q15)
		Intragenic	t(1;11)(p31;q25),**inv(12)(p12q14)**
		Intragenic	del(12)(q15q23)
		Intragenic	inv(12)(p11.2q15)
		Intragenic	inv(12)(q15q?)

		Intragenic	ins(12;12)(q15;q13q15),?inv(12)(p?q?)
		Intragenic	der(2)(2pter–.2q37::3q?–.3q?::12q15–.12qter), del(3)(q21),**der(12)del(12) (q15)?inv(12)(p?q?)**
		Intragenic	inv(12)?(q15q24.1)
		Intragenic	del(12)(q15q21.2)
		Intragenic	del(12)(q13.1q21.2)
		Intragenic	inv(12)(p12q15)
		Intragenic	inv(12)(q15q22)
		Intragenic	der(12)del(12)(q13q15)inv(12)(p11.2 or p12q21)
		Intragenic	inv(9)(p13q13),**inv(12)(q15q22)**
		Intragenic	ins(12;12)(q15;q15q22)
		Intragenic	inv(12)(q?q?)
		Intragenic	der(12)inv(12)(p11.2q15)inv(12)(q15p13)
		Intragenic	t(4;11)(p16;q23),**inv(12)(p11.2 or p12q15)**
		Intragenic	ins(12;12)(q15;q15q21)
		Intragenic	t(4;11)(p16;q23),inv(12)(p11.2 or p12q15)
	[55]	Intragenic	inv(12)(q14q21-23)
		13	del(6)(q24),**t(12;13)(q15;q13)**
		14	t(12;14)(q15;q24)
		14	der(14)t(12;14)(q15;q24)
		14	der(14)ins(14;12)(q24;q13q24.3)inv(12)(q15q?)
		14	ins(14;12)(q24;q13q24.1),inv(12)(?)
		14	ins(14;12)(q24;q15q24.1)
		14	add(11)(p?),**der(12)inv(12)(?)t(12;14),der(14)t(12;14)**
		14	+8,+8,t(12;14)(q15;q24)
		14	inv(7)(?),**t(12;14)(q15;q24)**
		14	add(1)(p32),del(3)(p21),**t(12;14)(q15;q21)1mar**
		14	der(12)t(12;14)(q15;q22),der(14)t(12;14)(q15;q12 or q13)
		14	ins(12;14)(q15;q22q24)
		16	**t(12;12;16)(p12;q15;p12 or p13.1)**,del(22)(q12 or q13)
		16	ins(16;12)(p13.1;q24.1q15)
		17	inv(6)(p12q21),**der(12)t(12;17)(p12;q23)del(12)(q15), der(17)t(12;17)(q15;q23)**
		18	der(12)inv(12)(q14q22)t(12;18)(q24.1;q21),der(18)t(12;18)(q24.1;q21)
		20	der(9)inv(9)(p13q13)del(9)(q22),**t(12;20)(q15;q13.2 or q13.3)**
		X	ins(X;12)(p22.1;q13q15)
		X	t(X;12)(q26;q15)
		X	t(X;12)(q24;q15)
		Y	ins(Y;12)(q12;q15q24.1)
		Unknown	ins(12;?)(q15;?)
		9, 16	t(3;6)(p25 or p26;p21.1 or p21.2),**der(9)t(9;12)(q34;q15),der(12)t(12;16) (q15;q21)**,del(16)(q21)
		11, 15, 17	der(11)t(11;17)(q14;p11.2),**der(12)t(11;12)(q14;q15), ins(15;12)(q22;q15q22), der(17)t(12;17)(q22;p11.2)**

		18, 20	der(3)del(3)(p11)del(3)(q23), **der(12)t(12;20)(q15;p11.2),**der(18)inv(18)(p13q21) **t(12;18)(q15;q21),** der(20)(3pter–.3p11::3q23–.37q29::20p11.2–.20qter)
		3, 10, 11	del(3)(q25),der(10)t(10;18)(p11.2;q21.3)**t(10;12)(q11.2;q15), der(12)t(10;12) (p11.2;p12)t(3;12)(q25;q15)/idem,t(11;12)(q21;q13)**
		13, 17	del(6)(p21.1),**der(12)t(12;13)(p12 or p13;q12)t(12;17)(q15;p13),der(13)t(12;13) (q15;q12)**, der(17)t(6;17)(p21.1;p13)
		5, 9	t(5;7)(p15.1;q32),der(6)inv(6)?,t(9;12)(p22;q15)
		7, 9	del(5)(q13q31),**t(7;12;19)(p14;q13;q13.4)**
		Y, 17	t(Y;12;17)
		13, 18	-12,**der(13)t(12;13)(q13;q12)**,del(14)(q22q24), **der(18)t(12;18)(p11.2;p11.1)**
Well-differentiated Liposarcoma	[39]	4	+der(4;12)
		1, 4, 10, 15	+r(1;4;10;12;15)
		1, 4, 10, 15	+der(1;4;10;12;15)
		11	+r(11;12)
		13	+r(12;13)
		1	+r(1;12)
		1	+der(1;12)
		Intragenic	+r(12)
		Intragenic	+der(12)

References

1. Bartuma, H.; Hallor, K. H.; Panagopoulos, I.; Collin, A.; Rydholm, A.; Gustafson, P.; Bauer, H. C.; Brosjo, O.; Domanski, H. A.; Mandahl, N.; Mertens, F., Assessment of the clinical and molecular impact of different cytogenetic subgroups in a series of 272 lipomas with abnormal karyotype. *Genes, chromosomes & cancer* **2007,** 46, (6), 594-606.
2. Bartuma, H.; Panagopoulos, I.; Collin, A.; Trombetta, D.; Domanski, H. A.; Mandahl, N.; Mertens, F., Expression levels of HMGA2 in adipocytic tumors correlate with morphologic and cytogenetic subgroups. *Molecular cancer* **2009,** 8, 36-36.
3. Bianchini, L.; Birtwisle, L.; Saada, E.; Bazin, A.; Long, E.; Roussel, J. F.; Michiels, J. F.; Forest, F.; Dani, C.; Myklebost, O.; Birtwisle-Peyrottes, I.; Pedeutour, F., Identification of PPAP2B as a novel recurrent translocation partner gene of HMGA2 in lipomas. *Genes, chromosomes & cancer* **2013,** 52, (6), 580-90.
4. Sreekantaiah, C.; Leong, S. P.; Karakousis, C. P.; McGee, D. L.; Rappaport, W. D.; Villar, H. V.; Neal, D.; Fleming, S.; Wankel, A.; Herrington, P. N.; et al., Cytogenetic profile of 109 lipomas. *Cancer Res* **1991,** 51, (1), 422-33.
5. Broberg, K.; Zhang, M.; Strombeck, B.; Isaksson, M.; Nilsson, M.; Mertens, F.; Mandahl, N.; Panagopoulos, I., Fusion of RDC1 with HMGA2 in lipomas as the result of chromosome aberrations involving 2q35-37 and 12q13-15. *Int J Oncol* **2002,** 21, (2), 321-6.
6. HATANO, H.; MORITA, T.; OGOSE, A.; HOTTA, T.; KOBAYASHI, H.; SEGAWA, H.; UCHIYAMA, T.; TAKENOUCHI, T.; SATO, T., Clinicopathological Features of Lipomas with Gene Fusions Involving HMGA2. *Anticancer Research* **2008,** 28, (1B), 535-538.
7. Turc-Carel, C.; Dal Cin, P.; Rao, U.; Karakousis, C.; Sandberg, A. A., Cytogenetic studies of adipose tissue tumors. I. A benign lipoma with reciprocal translocation t(3;12)(q28;q14). *Cancer Genet Cytogenet* **1986,** 23, (4), 283-9.
8. Dal Cin, P.; Turc-Carel, C.; Sandberg, A. A., Consistent involvement of band 12q14 in two different translocations in three lipomas from the same patient. *Cancer Genet Cytogenet* **1988,** 31, (2), 237-40.
9. Schoenmakers, E. F.; Wanschura, S.; Mols, R.; Bullerdiek, J.; Van den Berghe, H.; Van de Ven, W. J., Recurrent rearrangements in the high mobility group protein gene, HMGI-C, in benign mesenchymal tumours. *Nat Genet* **1995,** 10, (4), 436-44.
10. Ashar, H. R.; Fejzo, M. S.; Tkachenko, A.; Zhou, X.; Fletcher, J. A.; Weremowicz, S.; Morton, C. C.; Chada, K., Disruption of the architectural factor HMGI-C: DNA-binding AT hook motifs fused in lipomas to distinct transcriptional regulatory domains. *Cell* **1995,** 82, (1), 57-65.
11. Bartuma, H.; Nord, K. H.; Macchia, G.; Isaksson, M.; Nilsson, J.; Domanski, H. A.; Mandahl, N.; Mertens, F., Gene expression and single nucleotide polymorphism array analyses of spindle cell lipomas and conventional lipomas with 13q14 deletion. *Genes, chromosomes & cancer* **2011,** 50, (8), 619-32.
12. Petit, M. M. R.; Swarts, S.; Bridge, J. A.; Van de Ven, W. J. M., Expression of Reciprocal Fusion Transcripts of the HMGIC and LPP Genes in Parosteal Lipoma. *Cancer Genetics and Cytogenetics* **1998,** 106, (1), 18-23.
13. Nilsson, M.; Mertens, F.; Hoglund, M.; Mandahl, N.; Panagopoulos, I., Truncation and fusion of HMGA2 in lipomas with rearrangements of 5q32-->q33 and 12q14-->q15. *Cytogenet Genome Res* **2006,** 112, (1-2), 60-6.
14. Italiano, A.; Ebran, N.; Attias, R.; Chevallier, A.; Monticelli, I.; Mainguene, C.; Benchimol, D.; Pedeutour, F., NFIB rearrangement in superficial, retroperitoneal, and colonic lipomas with aberrations involving chromosome band 9p22. *Genes, chromosomes & cancer* **2008,** 47, (11), 971-7.
15. Lacaria, M.; El Demellawy, D.; McGowan-Jordan, J., A rare case of pediatric lipoma with t(9;12)(p22;q14) and evidence of HMGA2-NFIB gene fusion. *Cancer Genet* **2017,** 216-217, 100-104.
16. Pierron, A.; Fernandez, C.; Saada, E.; Keslair, F.; Hery, G.; Zattara, H.; Pedeutour, F., HMGA2-NFIB fusion in a pediatric intramuscular lipoma: a novel case of NFIB alteration in a large deep-seated adipocytic tumor. *Cancer Genet Cytogenet* **2009,** 195, (1), 66-70.
17. Nilsson, M.; Panagopoulos, I.; Mertens, F.; Mandahl, N., Fusion of the HMGA2 and NFIB genes in lipoma. *Virchows Arch* **2005,** 447, (5), 855-8.
18. Kazmierczak, B.; Dal Cin, P.; Wanschura, S.; Bartnitzke, S.; Van den Berghe, H.; Bullerdiek, J., Cloning and molecular characterization of part of a new gene fused to HMGIC in mesenchymal tumors. *The American journal of pathology* **1998,** 152, (2), 431-435.
19. Gisselsson, D.; Hoglund, M.; Mertens, F.; Mitelman, F.; Mandahl, N., Chromosomal organization of amplified chromosome 12 sequences in mesenchymal tumors detected by fluorescence in situ hybridization. *Genes, chromosomes & cancer* **1998,** 23, (3), 203-12.
20. Panagopoulos, I.; Gorunova, L.; Bjerkehagen, B.; Lobmaier, I.; Heim, S., The recurrent chromosomal translocation t(12;18)(q14~15;q12~21) causes the fusion gene HMGA2-SETBP1 and HMGA2 expression in lipoma and osteochondrolipoma. *International journal of oncology* **2015,** 47, (3), 884-890.
21. Petit, M. M. R.; Schoenmakers, E. F. P. M.; Huysmans, C.; Geurts, J. M. W.; Mandahl, N.; Van de Ven, W. J. M., LHFP,a Novel Translocation Partner Gene ofHMGICin a Lipoma, Is a Member of a New Family ofLHFP-like Genes. *Genomics* **1999,** 57, (3), 438-441.

22. Fejzo, M. S.; Ashar, H. R.; Krauter, K. S.; Powell, W. L.; Rein, M. S.; Weremowicz, S.; Yoon, S.-J.; Kucherlapati, R. S.; Chada, K.; Morton, C. C., Translocation breakpoints upstream of the HMGIC gene in uterine leiomyomata suggest dysregulation of this gene by a mechanism different from that in lipomas. *Genes, Chromosomes and Cancer* **1996,** 17, (1), 1-6.
23. Nilbert, M.; Heim, S.; Mandahl, N.; Flodérus, U. M.; Willén, H.; Mitelman, F., Characteristic chromosome abnormalities, including rearrangements of 6p, del(7q), +12, and t(12;14), in 44 uterine leiomyomas. *Human genetics* **1990,** 85, (6), 605-611.
24. Velagaleti, G. V. N.; Tonk, V. S.; Hakim, N. M.; Wang, X.; Zhang, H.; Erickson-Johnson, M. R.; Medeiros, F.; Oliveira, A. M., Fusion of HMGA2 to COG5 in uterine leiomyoma. *Cancer Genetics and Cytogenetics* **2010,** 202, (1), 11-16.
25. Kurose, K.; Mine, N.; Doi, D.; Ota, Y.; Yoneyama, K.; Konishi, H.; Araki, T.; Emi, M., Novel gene fusion of COX6C at 8q22–23 to HMGIC at 12q15 in a uterine leiomyoma. *Genes, Chromosomes and Cancer* **2000,** 27, (3), 303-307.
26. Mine, N.; Kurose, K.; Nagai, H.; Doi, D.; Ota, Y.; Yoneyama, K.; Konishi, H.; Araki, T.; Emi, M., Gene fusion involving HMGIC is a frequent aberration in uterine leiomyomas. *Journal of human genetics* **2001,** 46, (7), 408-412.
27. Polito, P.; Dal Cin, P.; Kazmierczak, B.; Rogalla, P.; Bullerdiek, J.; Van den Berghe, H., Deletion of HMG17 in uterine leiomyomas with ring chromosome 1. *Cancer Genet Cytogenet* **1999,** 108, (2), 107-9.
28. Kazmierczak, B.; Pohnke, Y.; Bullerdiek, J., Fusion transcripts between the HMGIC gene and RTVL-H-related sequences in mesenchymal tumors without cytogenetic aberrations. *Genomics* **1996,** 38, (2), 223-226.
29. Quade, B. J.; Weremowicz, S.; Neskey, D. M.; Vanni, R.; Ladd, C.; Dal Cin, P.; Morton, C. C., Fusion transcripts involving HMGA2 are not a common molecular mechanism in uterine leiomyomata with rearrangements in 12q15. *Cancer Res* **2003,** 63, (6), 1351-8.
30. Takahashi, T.; Nagai, N.; Oda, H.; Ohama, K.; Kamada, N.; Miyagawa, K., Evidence for RAD51L1/HMGIC fusion in the pathogenesis of uterine leiomyoma. *Genes, Chromosomes and Cancer* **2001,** 30, (2), 196-201.
31. Mine, N.; Kurose, K.; Konishi, H.; Araki, T.; Nagai, H.; Emi, M., Fusion of a sequence from HEI10 (14q11) to the HMGIC gene at 12q15 in a uterine leiomyoma. *Japanese journal of cancer research : Gann* **2001,** 92, (2), 135-139.
32. Gattas, G. J.; Quade, B. J.; Nowak, R. A.; Morton, C. C., HMGIC expression in human adult and fetal tissues and in uterine leiomyomata. *Genes, chromosomes & cancer* **1999,** 25, (4), 316-22.
33. Gross, K. L.; Panhuysen, C. I.; Kleinman, M. S.; Goldhammer, H.; Jones, E. S.; Nassery, N.; Stewart, E. A.; Morton, C. C., Involvement of fumarate hydratase in nonsyndromic uterine leiomyomas: genetic linkage analysis and FISH studies. *Genes, chromosomes & cancer* **2004,** 41, (3), 183-90.
34. Hennig, Y.; Rogalla, P.; Wanschura, S.; Frey, G.; Deichert, U.; Bartnitzke, S.; Bullerdiek, J., HMGIC expressed in a uterine leiomyoma with a deletion of the long arm of chromosome 7 along with a 12q14-15 rearrangement but not in tumors showing del(7) as the sole cytogenetic abnormality. *Cancer genetics and cytogenetics* **1997,** 96, (2), 129-133.
35. Pedeutour, F.; Deville, A.; Steyaert, H.; Ranchere-Vince, D.; Ambrosetti, D.; Sirvent, N., Rearrangement of HMGA2 in a case of infantile lipoblastoma without Plag1 alteration. *Pediatric blood & cancer* **2012,** 58, (5), 798-800.
36. Dahlén, A.; Mertens, F.; Rydholm, A.; Brosjö, O.; Wejde, J.; Mandahl, N.; Panagopoulos, I., Fusion, Disruption, and Expression of HMGA2 in Bone and Soft Tissue Chondromas. *Modern Pathology* **2003,** 16, (11), 1132-1140.
37. Andrieux, J.; Demory, J.-L.; Dupriez, B.; Quief, S.; Plantier, I.; Roumier, C.; Bauters, F.; Laï, J. L.; Kerckaert, J.-P., Dysregulation and overexpression of HMGA2 in myelofibrosis with myeloid metaplasia. *Genes, Chromosomes and Cancer* **2004,** 39, (1), 82-87.
38. Kazmierczak, B.; Dal Cin, P.; Sciot, R.; Van den Berghe, H.; Bullerdiek, J., Inflammatory myofibroblastic tumor with HMGIC rearrangement. *Cancer genetics and cytogenetics* **1999,** 112, (2), 156-160.
39. Pedeutour, F.; Forus, A.; Coindre, J.-M.; Berner, J.-M.; Nicolo, G.; Michiels, J.-F.; Terrier, P.; Ranchere-Vince, D.; Collin, F.; Myklebost, O.; Turc-Carel, C., Structure of the supernumerary ring and giant rod chromosomes in adipose tissue tumors. *Genes, Chromosomes and Cancer* **1999,** 24, (1), 30-41.
40. Hu, X.; Wang, Q.; Tang, M.; Barthel, F.; Amin, S.; Yoshihara, K.; Lang, F. M.; Martinez-Ledesma, E.; Lee, S. H.; Zheng, S.; Verhaak, R. G. W., TumorFusions: an integrative resource for cancer-associated transcript fusions. *Nucleic acids research* **2018,** 46, (D1), D1144-D1149.
41. Panagopoulos, I.; Gorunova, L.; Agostini, A.; Lobmaier, I.; Bjerkehagen, B.; Heim, S., Fusion of the HMGA2 and C9orf92 genes in myolipoma with t(9;12)(p22;q14). *Diagn Pathol* **2016,** 11, 22-22.
42. Kazmierczak, B.; Wanschura, S.; Meyer-Bolte, K.; Caselitz, J.; Meister, P.; Bartnitzke, S.; Van de Ven, W.; Bullerdiek, J., Cytogenic and molecular analysis of an aggressive angiomyxoma. *The American journal of pathology* **1995,** 147, (3), 580-585.

43. Rabban, J. T.; Dal Cin, P.; Oliva, E., HMGA2 rearrangement in a case of vulvar aggressive angiomyxoma. *Int J Gynecol Pathol* **2006,** 25, (4), 403-7.
44. Rawlinson, N. J.; West, W. W.; Nelson, M.; Bridge, J. A., Aggressive angiomyxoma with t(12;21) and HMGA2 rearrangement: report of a case and review of the literature. *Cancer Genetics and Cytogenetics* **2008,** 181, (2), 119-124.
45. Nucci, M. R.; Weremowicz, S.; Neskey, D. M.; Sornberger, K.; Tallini, G.; Morton, C. C.; Quade, B. J., Chromosomal translocation t(8;12) induces aberrant HMGIC expression in aggressive angiomyxoma of the vulva. *Genes, chromosomes & cancer* **2001,** 32, (2), 172-6.
46. Medeiros, F.; Erickson-Johnson, M. R.; Keeney, G. L.; Clayton, A. C.; Nascimento, A. G.; Wang, X.; Oliveira, A. M., Frequency and characterization of HMGA2 and HMGA1 rearrangements in mesenchymal tumors of the lower genital tract. *Genes, chromosomes & cancer* **2007,** 46, (11), 981-90.
47. Cokelaere, K.; Debiec-Rychter, M.; De Wolf-Peeters, C.; Hagemeijer, A.; Sciot, R., Hyaline vascular Castleman's disease with HMGIC rearrangement in follicular dendritic cells: molecular evidence of mesenchymal tumorigenesis. *The American journal of surgical pathology* **2002,** 26, (5), 662-9.
48. Broberg, K.; Hoglund, M.; Limon, J.; Lindstrand, A.; Toksvig-Larsen, S.; Mandahl, N.; Mertens, F., Rearrangement of the neoplasia-associated gene HMGIC in synovia from patients with osteoarthritis. *Genes, chromosomes & cancer* **1999,** 24, (3), 278-82.
49. Panagopoulos, I.; Bjerkehagen, B.; Gorunova, L.; Taksdal, I.; Heim, S., Rearrangement of chromosome bands 12q14~15 causing HMGA2-SOX5 gene fusion and HMGA2 expression in extraskeletal osteochondroma. *Oncol Rep* **2015,** 34, (2), 577-84.
50. Panagopoulos, I.; Bjerkehagen, B.; Gorunova, L.; Taksdal, I.; Heim, S., Rearrangement of chromosome bands 12q14~15 causing HMGA2-SOX5 gene fusion and HMGA2 expression in extraskeletal osteochondroma. *Oncology reports* **2015,** 34, (2), 577-584.
51. Panagopoulos, I.; Bjerkehagen, B.; Gorunova, L.; Berner, J. M.; Boye, K.; Heim, S., Several fusion genes identified by whole transcriptome sequencing in a spindle cell sarcoma with rearrangements of chromosome arm 12q and MDM2 amplification. *Int J Oncol* **2014,** 45, (5), 1829-36.
52. Van Dorpe, J.; Dal Cin, P.; Weremowicz, S.; Van Leuven, F.; de Wever, I.; Van den Berghe, H.; Fletcher, C. D.; Sciot, R., Translocation of the HMGI-C (HMGA2) gene in a benign mesenchymoma (chondrolipoangioma). *Virchows Arch* **2002,** 440, (5), 485-90.
53. Kazmierczak, B.; Meyer-Bolte, K.; Tran, K. H.; Wöckel, W.; Breightman, I.; Rosigkeit, J.; Bartnitzke, S.; Bullerdiek, J., A high frequency of tumors with rearrangements of genes of the HMGI(Y) family in a series of 191 pulmonary chondroid hamartomas. *Genes, Chromosomes and Cancer* **1999,** 26, (2), 125-133.
54. Rogalla, P.; Kazmierczak, B.; Meyer-Bolte, K.; Tran, K. H.; Bullerdiek, J., The t(3;12)(q27;q14-q15) with underlying HMGIC-LPP fusion is not determining an adipocytic phenotype. *Genes, Chromosomes and Cancer* **1998,** 22, (2), 100-104.
55. Wanschura, S.; Cin, P. D.; Kazmierczak, B.; Bartnitzke, S.; Van den Berghe, H.; Bullerdiek, J., Hidden paracentric inversions of chromosome arm 12q affecting the HMGIC gene. *Genes, Chromosomes and Cancer* **1997,** 18, (4), 322-323.
